# ManChEWS: Royal Manchester Children's Hospital early warning score

**DOI:** 10.1186/cc9927

**Published:** 2011-03-11

**Authors:** V Joshi, R Barber, R Yates

**Affiliations:** 1 Royal Manchester Children's Hospital, Manchester, UK

## Introduction

Unrecognised clinical deterioration resulting in near or actual cardiorespiratory arrest in hospitalised children sadly still occurs. The majority of these events may be preventable. Royal Manchester Children's Hospital (RMCH) introduced a simple track and trigger Early Warning System (ManChEWS) in 2005 by which variation in six key physiological parameters is scored according to a traffic-light system in routine nursing observations. The aim was to evaluate use of ManChEWS since its introduction, in order to allow continued improvement and development.

## Methods

Three audits were carried out: an audit to evaluate ManChEWS in emergency admissions to the PICU or PHDU (2006 to 2007), a prospective audit of children who trigger EWS on the ward but do not require admission to the PHDU/PICU (2009), and an audit to evaluate the use of ManChEWS in children that died between 2005 and 2008 following an acute deterioration on the wards.

## Results

ManChEWS correctly identifies the clinically deteriorating child on the ward. ManChEWS is over-triggering, leading to staff becoming immune to triggers. This is due to the high frequency of underlying illness in children admitted to RMCH. Medical staff are not currently redefining parameters for children with abnormal baseline parameters. ManChEWS is not being universally used in RMCH. Twenty-five per cent of deaths in RMCH were attributable in part to 'the failure to recognise a sick child'. These might have been prevented by the correct use of ManChEWS.

## Conclusions

ManChEWS correctly identifies the deteriorating child and offers staff a clear pathway for escalation of care and senior review. ManChEWS is not being used correctly on the wards by medical or nursing staff. For patients with underlying disease, ManChEWS over-triggers, leading to staff becoming immune to triggers. *Developments and the future *Development of an EWS Steering Group. Daily review of patients triggering ManChEWS by development of an outreach team. Electronic EWS implementation across the Trust. Patients with underlying illness may have individualised parameters set by senior medical staff.

